# Balance control strategies during perturbed and unperturbed balance in standing and handstand

**DOI:** 10.1098/rsos.161018

**Published:** 2017-07-26

**Authors:** Glen M. Blenkinsop, Matthew T. G. Pain, Michael J. Hiley

**Affiliations:** School of Sport, Exercise and Health Sciences, Loughborough University, Loughborough, Leicestershire LE11 3TU, UK

**Keywords:** postural control, ankle strategy, wrist strategy

## Abstract

Insights into sensorimotor control of balance were examined by the assessment of perturbed and unperturbed balance in standing and handstand postures. During perturbed and unperturbed balance in standing, the most prevalent control strategy was an ankle strategy, which was employed for more than 90% of the time in balance. During perturbed and unperturbed balance in handstand, the most prevalent control strategy was a wrist strategy, which was employed for more than 75% of the time in balance. In both postures, these strategies may be described as a single segment inverted pendulum control strategy, where the multi-segment system is controlled by torque about the most inferior joint with compensatory torques about all superior joints acting in the same direction to maintain a fixed orientation between superior segments. In contrast to previous literature, surprisingly little time was spent in a mixed strategy, representing less than 1% of time in standing balance and approximately 2% of time in handstand balance. Findings indicate that although the central nervous system may employ a number of control strategies during a trial, these strategies are employed individually rather than simultaneously.

## 1. Introduction

Two popular approaches for studying postural control are the perturbation and the non-perturbation paradigms [1]. The concept of postural control strategies emerged from investigations using the perturbation paradigm to describe distinct muscle synergies in response to platform perturbations [2–4]. During upright stance, platform perturbations elicit relatively stereotypical patterns of leg and trunk muscle activation with EMG latencies of approximately 65–130 ms [3,5,6]. The postural control system, rather than varying the pattern of muscular contractions continuously, uses distinct strategies within bounded regions of the position space, such as the ankle and hip strategies [2]. The ankle strategy involves delayed activation of the ankle, thigh and trunk muscles radiating distally to proximally on the same dorsal or ventral aspect of the body [2,3]. The hip strategy involves the delayed activation of the trunk and thigh muscles, radiating in a proximal-to-distal fashion. For example, in response to a posterior movement of the support surface the ankle strategy would result in activation of the ankle plantar-flexors, knee flexors and hip extensors, while the hip strategy would result in activation of the knee extensors and hip flexors [2,3].

Mechanically, the ankle strategy consists of rotation of the body about the ankle joint with minimal movement about superior joints [2], allowing the body to act as a single-segment inverted pendulum controlled by ankle joint torque. A hip strategy involves the upper body rotating forward and downward, imposing a backward rotation on the lower body while also decreasing the moment of inertia about the ankle and allowing a given ankle torque to effect a higher angular acceleration of the body [7]. The ankle strategy is limited by the foot's ability to exert torque in contact with the support surface, whereas the hip strategy is limited by surface friction and the ability to produce horizontal force against the support surface [2,3]. Consequently, the ankle strategy is expected to be employed for unperturbed stance and for slow and low amplitude perturbations, whereas the hip strategy is expected to be employed for fast or large amplitude perturbations, or when the support surface is narrow and little ankle torque can be applied [2,3].

Control strategies may be identified by measuring kinematics, kinetics or muscle activity [2–4,7,8], creating ambiguity about which variables are most appropriate for defining ankle and hip strategies [9,10]. Further confusion exists regarding the correct definitions of ankle and hip strategies, and whether or not a ‘pure’ strategy is possible in either case [2–4,7–11]. For example, Runge *et al.* [7] suggest the presence of hip torques and knee and hip joint motion for slow perturbation velocities could indicate a mixed strategy was used to maintain balance. Similarly, Colobert *et al.* [11] conclude that the presence of hip motion, greater than ankle motion, during a forced ankle strategy suggests that balance is maintained using multiple strategies. Alternatively, Nashner & McCollum [2] and Horak & Nashner [3] describe the ankle strategy as containing small amounts of hip rotation with the majority of joint rotation occurring about the ankle. Regardless of which strategy is employed by the central nervous system (CNS), motion and torque about both the ankle and hip is inevitable, as accelerations of one segment will result in accelerations imposed on other segments that must be either resisted or assisted by the appropriate musculature [2,7,8,12]. Ultimately, an attempt at an ankle strategy will require compensatory hip torque acting in the same direction as ankle torque to resist the load imposed on it by the acceleration of the legs. Conversely, an attempt at a hip strategy will require complementary ankle torque acting in the opposite direction to hip torque to achieve the required anti-phase rotation of the upper and lower body. The two strategies may therefore be identified by assessing the direction of hip torque relative to the direction of ankle torque, and may be distinguished using correlation analysis [13].

Responses to discrete perturbations result in discrete response synergies; however, during unperturbed stance numerous corrections are implemented in an attempt to remain upright. Although examining how subjects respond to controlled disturbances can provide useful insights into sensorimotor control of balance, responses to perturbations reveal only one aspect of the postural control system [4]. Only examining the discrete time period shortly after a disturbance may not provide all relevant information on how humans attempt to maintain balance during varying tasks. Spectral analysis of joint kinematics during longer duration trials reveal that balance can be described as a multi-link pendulum with ankle and hip strategies viewed as ‘simultaneous coexisting excitable modes', both always present, but one which may predominate depending upon the characteristics of the available sensory information, task or perturbation [14]. Time-frequency analysis of centre of pressure motion during unperturbed stance has revealed that human balance is non-stationary with time-varying properties [15,16]. Gurses *et al.* [17] examined the evolution of intersegmental coordination over time, adapting the model of Kuo *et al.* [18] by dividing trials into overlapping time segments of 16 s duration. Results indicated statistical and spectral characteristics across the entire trial can differ from individual time segments of the same trial displaying time-varying intersegmental coordination behaviours [17]. Employing stationary-based data analysis techniques to non-stationary balance or coordination data will result in misrepresentations of the full temporal characteristics of postural control. Using correlations of ankle and hip joint torques with moving windows may provide a suitable means to study the time-varying behaviour of postural control strategies.

Control strategies may be described as emergent neural control processes which may be best differentiated by what the CNS is attempting to control [4]. Although ankle and hip strategies in standing balance have been identified, and some studies have described how one strategy may be preferred over another in different situations, the cause for this preference is less clear. Kuo [12] created a constrained model of human balance based on feasible acceleration sets to examine the cost of activating muscles to control posture. The model predicted that, for a given magnitude of horizontal acceleration, the hip strategy is most effective at controlling the centre of mass (COM) with minimal muscle activation [12,18]. In addition, the hip strategy is faster and can tolerate greater time latencies before becoming unstable [18]. The hip strategy appears to be more robust and more efficient than the ankle strategy, which might explain why it is used during perturbed conditions, but does not explain why it is not used during unperturbed conditions. However, for many goals, maximizing efficiency does not involve a simple minimization of effort [19]. Ricco & Stoffregen [19] explain how uncontrolled motion of the perception and action systems can hinder their function. For example, the increased head movement resulting from a hip strategy will require a larger compensatory eye movement than that resulting from an ankle strategy. This interaction between an organism and the environment, and specifically the goals of additional tasks being performed, can constrain which control strategy may be employed and may explain why an ankle strategy is preferred during unconstrained standing. What, therefore, would this suggest for balance control in postures other than upright standing? Examining various postures and discovering common invariant traits in the way control strategies are implemented could aid understanding of how the CNS attempts to control balance. Handstand balance performed by experienced gymnasts provides an alternative perspective to normal upright stance for understanding this complex system [20].

In handstand, the task is to keep the body in an inverted posture, supported by the hands, with anteroposterior motion primarily controlled by a wrist strategy, which can be considered equivalent to the ankle strategy in standing [13,21]. Furthermore, other control strategies can be called upon, with a hip strategy in standing being equivalent to a shoulder or hip strategy in handstand [13]. However, while in standing a hip strategy can result in large movements of the head, significantly affecting perception and action coupling, in the handstand posture the use of a shoulder or hip strategy results in very little movement of the head. Why then is the wrist strategy still dominant during unperturbed handstand balance, and how will this change during perturbed conditions? Therefore, the purpose of this study was to determine the prevalent control strategies attempted by individuals to retain balance in both handstand and standing postures during both perturbed and unperturbed balance tasks. It is hypothesized that the preferred control strategy in unperturbed balance is a single segment control strategy, with control about the ankle in standing and the wrist in handstand. During perturbed balance, it is hypothesized that this preferred control strategy will be substituted for one which can respond more quickly resulting in a hip strategy in standing and an elbow, shoulder or hip strategy in handstand.

## 2. Material and methods

### 2.1. Subjects

Twelve gymnasts experienced at balancing in handstand were recruited for this study, including nine males (age: 23.1 ± 3.6 (mean ± s.d.) years; mass: 69.9 ± 2.2 kg; height: 1.73 ± 0.05 m) and three females (age: 20.5 ± 0.7 years; mass: 57.9 ± 1.9 kg; height: 1.64 ± 0.02 m). All gymnasts had over 10 years’ experience of structured training and competitive gymnastics at national level, and could balance in the handstand position for at least 30 s while maintaining a static base of support. All gymnasts were free from injury during the testing period and gave written informed consent for participation in the study that was approved by the University Ethical Advisory Committee.

### 2.2. Procedure

Perturbed and unperturbed balance was assessed in two separate sessions approximately one week apart. Gymnasts completed unperturbed balance trials for a maximum of 30 s duration in both handstand and standing postures with eyes open and eyes closed conditions. Each condition was completed in a block of five trials with a minimum 1 min rest between each trial in one of the orders described in table 1. During all trials, gymnasts were instructed to maintain a static base of support, and attempt to remain in, or return to, the standard starting position of: fully extended arms, trunk and legs with feet together for handstand trials, and fully extended legs and trunk with arms by the side for standing trials. A change to the base of support, such as a shuffle or a step, was considered as a failure to maintain balance, and the trial was halted.

**Table 1.** Two orders of blocks of trials used for assessing unperturbed balance, to which gymnasts were randomly assigned.

order one	order two
handstand – eyes open	handstand – eyes closed
standing – eyes open	standing – eyes closed
standing – eyes closed	standing – eyes open
handstand – eyes closed	handstand – eyes open

**Figure 1.**
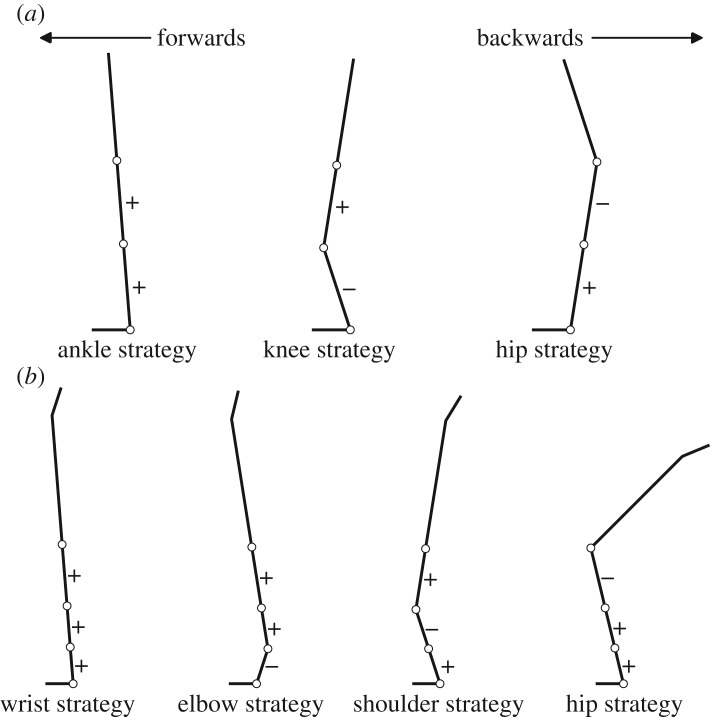
Examples of control strategies in (*a*) standing and (*b*) handstand, identified by positive and negative correlations of adjacent joint torques.

Perturbed balance was assessed via 12 randomized discrete platform translations in the eyes open condition for both handstand and standing postures, with three trials for each of four types of perturbation: backwards large, backwards small, forwards large and forwards small (see figure 1 for directions). Large perturbations had an amplitude of 0.1 m, peak velocity of ±0.2 m s^−1^ and peak acceleration of ±1.2 m s^−2^; small perturbations had an amplitude of 0.05 m, peak velocity of ±0.1 m s^−1^ and peak acceleration of ±1.2 m s^−2^. Data collection commenced once the gymnast was in a stable balanced position, with 1–3 s of static balance performed before the initiation of the perturbation. Perturbed trials were stopped when either the subject failed to maintain balance with a static base of support or when the experimenter judged the subject had regained balance and returned to the standard starting position.

### 2.3. Data collection

All balance trials were completed on a CAREN system (Motek Medical), with perturbations created by translation of a six degree of freedom Stewart platform controlled by a custom script written in the Motek Medical D-Flow software. Kinematic data were collected using nine T20 Vicon (Vicon, Oxford Metrics Group) cameras operating at 200 Hz. A marker set consisting of 53 spherical markers of 14 mm diameter was used to divide the body into 18 segments. Individual segmental inertial parameters were obtained via anthropometric measurements using the inertia model of Yeadon [22]. Kinetic data were collected via two 0.4 m × 0.6 m force plates (Bertec FP4060-07) with a sample frequency of 2000 Hz. Four additional markers were placed outside the area of the force plates to track the motion of the platform to correct for centre of pressure (COP) and inertial force errors introduced into the force plate measures [23].

### 2.4. Data processing

Force, moment and COP data were down-sampled to 200 Hz in Matlab before being combined with kinematic data for inverse dynamics calculations. Low-pass cut-off frequency estimates for force, COP displacements and marker displacements were 4–9 Hz based on residual analysis and 6–10 Hz based on power spectral analysis. To provide consistency across trials, and to reduce the possibility of attenuating the true signal by over-filtering, all force, COP and marker data were filtered using a fourth order, zero lag, low-pass Butterworth filter with a cut-off frequency of 10 Hz.

Kinematic data were used to calculate segment COM linear displacements and accelerations, and segment angular velocities and accelerations using quaternion algebra [24]. Kinematic, inertia and kinetic data were combined to calculate three-dimensional joint forces and moments using wrench notation [24]. All joint torque analysis was performed in the sagittal plane, therefore right and left joint moments were summed to provide a total joint moment about the global mediolateral axis for: ankle, knee, hip, shoulder, elbow and wrist joints.

### 2.5. Data analysis

Control strategies used to maintain balance in both postures were determined by multiple correlations of adjacent joint torques. In the handstand posture, Yeadon & Trewartha [13] described a wrist strategy via a planar two-segment system controlled by a wrist torque with synergistic hip torque acting in the same direction to maintain a fixed orientation between the trunk and thigh. By contrast, the hip strategy would involve a hip torque operating in the opposite direction to the wrist torque to produce the required anti-phase motion. Expanding this model into a planar multi-segment model of standing balance comprising a foot, shank, thigh and trunk, an ankle strategy would require an ankle torque with synergistic torques about the knee and hip acting in the same direction to maintain a fixed orientation between the segments above the ankle. An ankle strategy can therefore be identified by positive correlations between all adjacent joint torques (figure 1*a*). Consequently, other control strategies can be identified from a negative correlation between: ankle and knee torques (knee strategy), or knee and hip torques (hip strategy; figure 1*a*). Similarly, a wrist strategy in handstand balance can be identified by positive correlations between all adjacent joints from wrist, elbow, shoulder and hip joint torques (figure 1*b*), with other strategies identified by a negative correlation between: wrist and elbow torques (elbow strategy), elbow and shoulder torques (shoulder strategy) or shoulder and hip torques (hip strategy; figure 1*b*).

**Figure 2.**
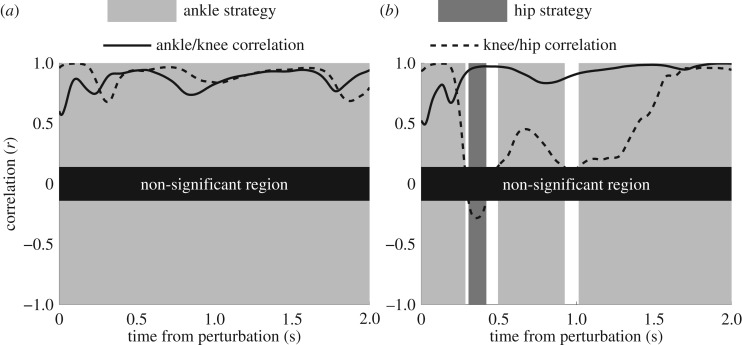
Examples of the time spent in different control strategies during (*a*) backwards and (*b*) forwards perturbations in standing.

**Figure 3.**
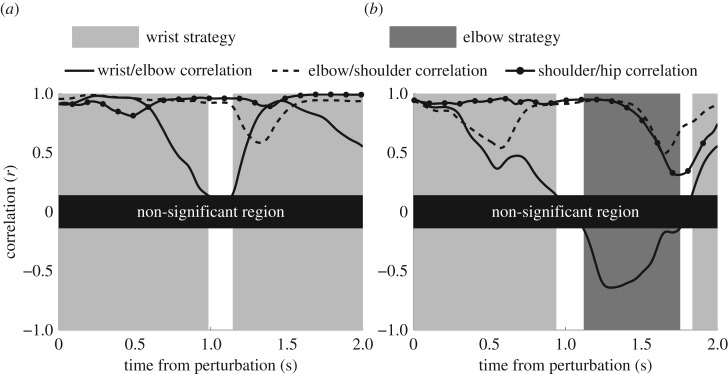
Examples of the time spent in different control strategies during (*a*) backwards and (*b*) forwards perturbations in handstand.

The amount of time spent using different control strategies was determined by performing multiple correlations of adjacent joint torques with a moving 1 s window over the full duration of the trial for unperturbed balance, and for a period of 1 s before to 2 s after the initiation of the perturbation for perturbed trials (figures 2 and 3). Correlations were implemented in Matlab for each 1 s window and the control strategy used was identified based on the descriptions above. The significance level for all correlations was set to 0.05, corresponding to an *R* value (Pearson product) of ±0.14 for 200 data points used for each one second window. A non-significant strategy was identified as the time when a control strategy could not be determined due to at least one non-significant correlation (figures 2 and 3), and a mixed strategy was determined by more than one negative correlation, indicating multiple control strategies were employed.

### 2.6. Statistical analysis

Prior to statistical analysis, trials were combined to create a mean score for each subject under each posture and condition. The four unperturbed conditions were: handstand with eyes open, handstand with eyes closed, standing with eyes open and standing with eyes closed. The eight perturbed conditions were standing and handstand postures in each of the four perturbations: forwards small, forwards large, backwards small and backwards large. Only the first three most prevalent control strategies were used for further analysis to allow a statistical comparison between standing (three possible strategies) and handstand (four possible strategies). Control strategies in the present study have been converted to a percentage of the trial time. Although necessary, this results in what is known as the constant sum problem, whereby different components of the data become dependent, introducing a negative bias into correlations [25,26]. Therefore, further analysis will focus mainly on the primary control strategy, and the reader is advised to be cautious with interpreting changes in other control strategies which are likely to be a consequence of the primary control strategy also changing.

Two separate two-way repeated measures ANOVAs were performed to assess the differences between the percentages of time spent in different control strategies. The first ANOVA assessed differences in unperturbed trials between posture (handstand versus standing) and vision (eyes open versus eyes closed). The second ANOVA assessed differences in perturbed trials between posture (handstand versus standing) and perturbation (backwards large, backwards small, forwards large, forwards small). Further comparisons were made using multiple repeated measures *t*-tests with a Bonferroni correction. Prior to statistical testing, all data were assessed for normality and sphericity by the one-sample Kolmogorov–Smirnov test and Mauchly's test of sphericity. A Greenhouse–Geisser correction was used to adapt the degrees of freedom of statistical tests for any data that was found to violate the assumption of sphericity. The significance level for all statistical tests was set to 0.05.

## 3. Results

During standing balance, the most prevalent control strategy employed was an ankle strategy, followed by a hip strategy, and a knee strategy was employed the least (table 2). During handstand balance, the most prevalent control strategy employed was a wrist strategy, followed by an elbow strategy, then a shoulder strategy, and a hip strategy was employed the least (table 3). To aid comparisons between standing and handstand postures, the first three most prevalent control strategies were ordered and identified as the primary, secondary and tertiary strategies based on the amount of time spent in each.

**Table 2.** The percentage of time spent in each control strategy during unperturbed and perturbed balance in standing.

	ankle	knee	hip	mixed	non-significant
	mean ± s.d.	mean ± s.d.	mean ± s.d.	mean ± s.d.	mean ± s.d.
unperturbed	95.2 ± 3.9	0.4 ± 0.5	1.4 ± 1.6	0.0 ± 0.1	3.0 ± 2.1
perturbed	90.6 ± 9.9	0.9 ± 2.3	2.2 ± 4.1	0.2 ± 1.0	6.0 ± 6.2

**Table 3.** The percentage of time spent in each control strategy during unperturbed and perturbed balance in handstand.

	wrist	elbow	shoulder	hip	mixed	non-significant
	mean ± s.d.	mean ± s.d.	mean ± s.d.	mean ± s.d.	mean ± s.d.	mean ± s.d.
unperturbed	88.3 ± 13.4	4.2 ± 5.3	0.6 ± 1.3	0.2 ± 0.3	2.0 ± 3.7	4.8 ± 5.0
perturbed	75.8 ± 18.4	10.8 ± 11.3	1.0 ± 3.9	0.9 ± 3.9	1.5 ± 4.4	10.1 ± 8.0

**Figure 4.**
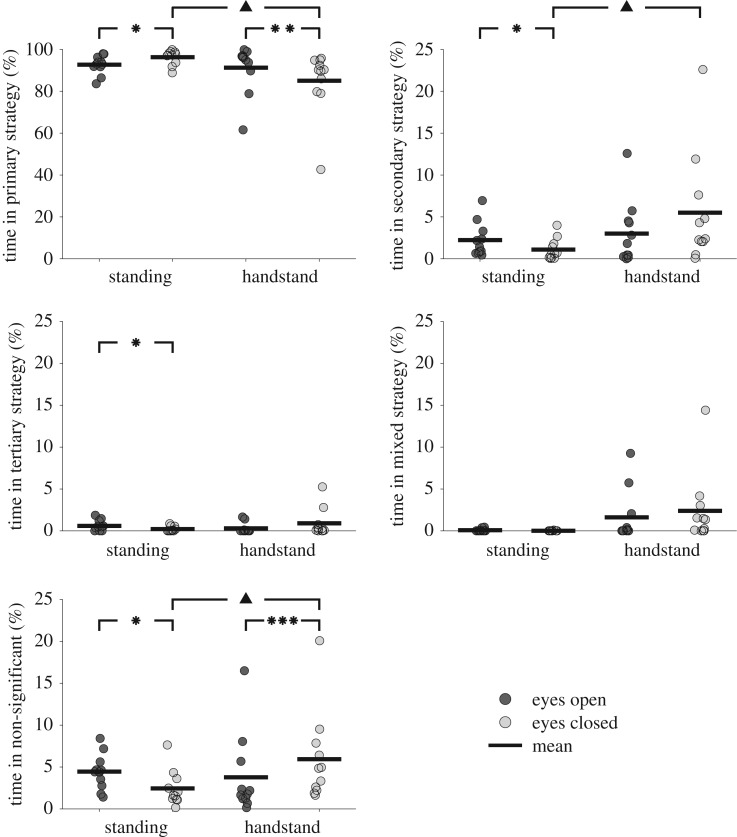
The mean time spent in each control strategy for unperturbed balance in standing and handstand in eyes open and eyes closed conditions (significant differences between standing and handstand are indicated by ▴ (*p* < 0.05); significant differences between eyes open and eyes closed are indicated by *(*p* < 0.05), **(*p* < 0.01) and ***(*p* < 0.005)).

During unperturbed balance, there was a statistically significant interaction between the effects of vision and posture on the percentage of time spent in a primary, secondary, tertiary and non-significant strategy. There were no significant differences between any comparisons for the percentage of time spent in a mixed strategy (figure 4). *Post hoc* analyses showed there were significant differences for the time spent in the primary and non-significant control strategies between standing and handstand balance with eyes closed, and between eyes open and eyes closed conditions in both standing and handstand balance (figure 4). In addition, significant differences were found for the time spent in the secondary control strategy between standing and handstand balance with eyes closed, and for the time spent in the secondary and tertiary control strategies between eyes open and eyes closed conditions in standing balance (figure 4). There were no significant differences in the percentage of time spent in any of the control strategies between standing and handstand balance with eyes open.

**Figure 5.**
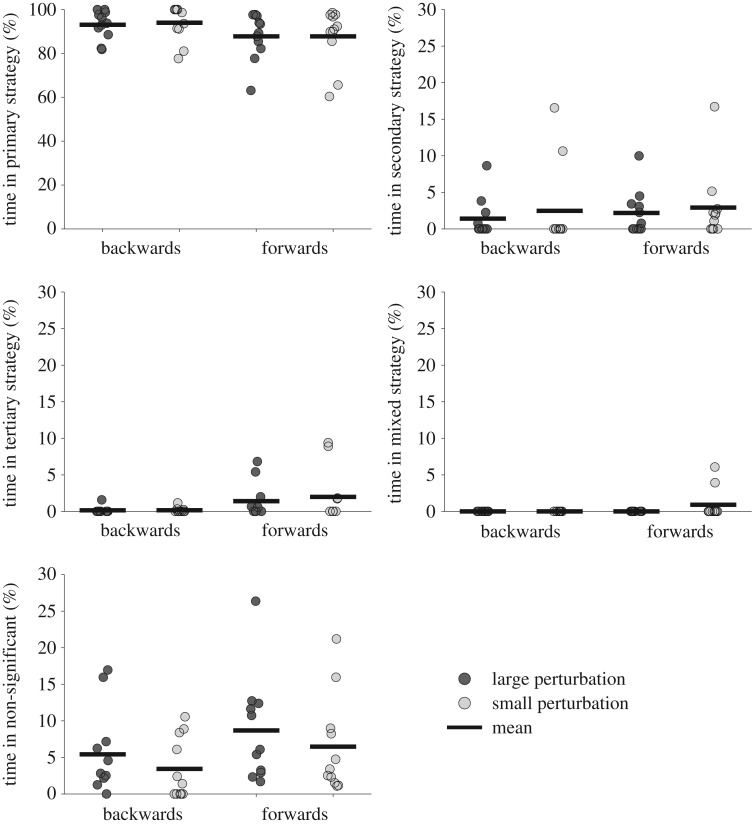
The mean time spent in each control strategy for perturbed balance in standing (no significant differences were found between perturbation directions or magnitudes).

**Figure 6.**
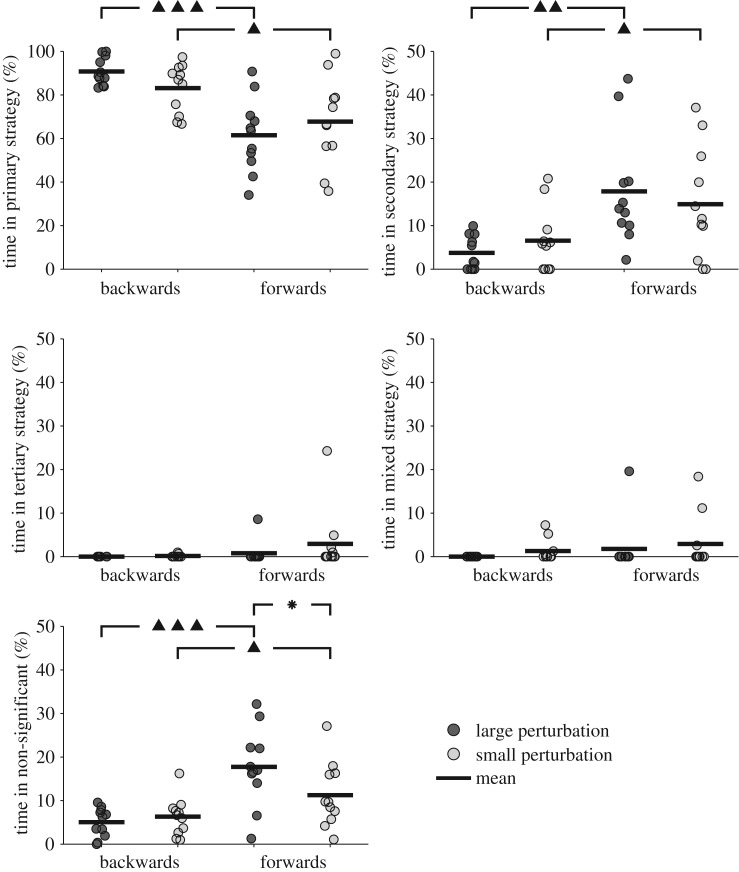
The mean time spent in each control strategy for perturbed balance in handstand (significant differences between perturbation directions are indicated by ▴(*p* < 0.05), ▴▴(*p* <* *0.01) and ▴▴▴(*p* < 0.005); significant differences between perturbation magnitudes are indicated by *(*p *<* *0.05)).

During perturbed balance, there was a statistically significant interaction between the effects of perturbation type and posture on the percentage of time spent in a primary and secondary control strategy (electronic supplementary material, tables S1 and S2). There were also statistically significant main effects for posture and perturbation type for the time spent in a non-significant control strategy. Once again there were no significant differences between any comparisons for the percentage of time spent in a mixed control strategy (figures 5 and 6). *Post hoc* analyses showed there were significant differences for the time spent in the primary control strategy between standing and handstand balance during forwards large, forwards small and backwards small perturbations. In addition, comparisons between standing and handstand balance showed significant differences were found for the time spent in the secondary control strategy during forwards small perturbations, and for the time spent in the secondary and non-significant control strategies for forwards large perturbations. During standing balance, there were no significant differences between perturbation directions or magnitudes for the time spent in any control strategy (figure 5). During handstand balance, there were significant differences for the time spent in the primary, secondary and non-significant control strategies between backwards large and forwards large perturbations, and between backwards small and forwards small perturbations (figure 6).

## 4. Discussion

Insights into sensorimotor control of balance can be achieved by studying how subjects respond to controlled disturbances; however, this may not be representative of balance during unperturbed conditions. Examining various postures during multiple tasks and discovering invariant traits in the way control strategies are implemented can aid understanding of how the CNS attempts to control balance. The aim of this study was to determine the percentage of time spent in different control strategies during perturbed and unperturbed balance in standing and handstand postures. It was hypothesized that the preferred control strategy in unperturbed balance would be a single segment control strategy, but during perturbed balance this would be substituted for a strategy that responds more quickly, such as a hip or shoulder strategy. It was found that the primary control strategy for both perturbed and unperturbed standing balance was an ankle strategy (table 2) and the primary control strategy for both perturbed and unperturbed handstand balance was a wrist strategy (table 3). Both strategies can be described as a single segment inverted pendulum control strategy, where the multi-segment system is controlled by torque about the most inferior joint with compensatory torques about all superior joints acting in the same direction to maintain a fixed orientation between segments.

During unperturbed standing balance, the ankle strategy was employed for approximately 95% of the time (table 2), supporting previous hypotheses that an ankle strategy is dominant when no external perturbations are present [2,3,8,12]. During unperturbed handstand balance, the wrist strategy was employed for approximately 88% of the time (table 3). Findings are in agreement with previous research which showed wrist joint torque was the dominant contributor to COM displacement [21] and is used to control COM displacement and velocity together with synergistic torques at the shoulder and hip [13]. Comparisons between unperturbed balance in standing and handstand revealed both postures use a single strategy for almost the entire time when balancing in both eyes open and eyes closed conditions. Removing vision resulted in contrary findings in standing and handstand trials, with standing displaying a significant increase, and handstand displaying a significant decrease, in the percentage of time spent in the primary control strategy. Consequently, these findings were accompanied with significant changes in the percentage of time spent in the secondary and non-significant trials, with a corresponding decrease during standing trials and an increase in handstand trials. The apparently contradictory finding between standing and handstand conditions may be explained by the difficulty of balancing in the two postures. Previous researchers have described standing balance as a biomechanically under-constrained task [27,28]. Although standing balance may be affected by a loss of vision, the amount of postural sway is considerably less during double leg stance without vision than in other postures with vision, such as toe stance [29], single leg stance [29,30] and handstand [30]. During under-constrained tasks, such as standing balance, there is a motor abundance available to the CNS to control COM motion [31]. The Uncontrolled Manifold hypothesis suggests that the CNS may relax elements of this control, allowing increased variance in aspects that will not have a direct effect on the performance of the task [32–34]. The increase in time spent in the primary control strategy during standing balance without vision may be attributed to the CNS tightening some of this control in an attempt to maintain task performance with reduced sensory input. Handstand balance, however, is not under-constrained, and is instead extremely challenging. The decrease in time spent in the primary control strategy during handstand without vision may represent the system struggling to deal with this challenging task, employing the secondary control strategy to assist, or even resulting in poor control with uncoordinated joint motion.

During perturbed standing balance, there were no significant differences between perturbation directions or magnitudes for the percentage of time spent in any control strategy (figure 5). In this study, it would appear that the range of perturbation magnitudes employed were not sufficient to elicit significantly different responses from the study population. Perturbations employed in this study were of a similar magnitude to previous research eliciting various control responses from ankle and hip strategies [2,3,7]. The lack of a significant difference here is probably because elite gymnasts were recruited, rather than average or clinical populations. During perturbed handstand balance, the largest differences between the percentage of time spent in different control strategies can be attributed to the perturbation direction rather than the magnitude of the perturbation (figure 6). Findings suggest that the constraints imposed by the direction of the perturbation interact with the biomechanical constraints of balancing in the handstand posture. Similar conclusions have been made with respect to standing balance [2,12], adding further support to the constraints-led approach proposed by Newell [35].

Analysis of 1 s moving windows of balance trials revealed that a single strategy is employed for almost the entire time. Although the time spent in different control strategies can vary between postures and conditions, there were no significant differences between any comparisons for the percentage of time spent in a mixed control strategy. During both perturbed and unperturbed balance the time spent in a mixed strategy amounted to less than 1% for standing trials (table 2) and approximately 2% for handstand trials (table 3). In contrast to previous research suggesting that mixed control strategies may be prevalent during balance [7,11], this study shows that the CNS is remarkably good at employing individual control strategies to maintain balance during a variety of tasks. Previous research indicating mixed strategies were employed during perturbed [7] or unperturbed [11] stance did not attempt to examine the time-varying aspects of balance, as was done in this study. A common problem with examining time-varying properties of data, such as using time-frequency estimators, is that time and frequency resolution cannot be increased simultaneously [15]. In this study, this limited the length of moving windows to a duration of 1 s, as the non-significant region (figures 2 and 3) would become too large otherwise. This was considered appropriate, as previous research has shown that 80% of the COP power spectral density is below 0.5 Hz for standing balance in young and elderly adults [36,37]. However, these analyses were based on the trajectory of the COP, whereas the analysis of the present study was based on torques at multiple joints. Frequency analysis of all joint torques showed that peak power spectral density and median frequencies occurred well below 0.5 Hz for all conditions. Consequently, the analysis of 1 s moving windows will identify the dominant control strategies during balance trials. Although some joint torques are likely to contain frequencies above the analysis resolution of 1 Hz, this will have minimal effect on the ability to detect control strategies, unless it is significantly higher than 1 Hz. Nevertheless, it is possible that a small number of control strategies were employed for very short periods of time and were not detected due to the limited time resolution. For example, it may be possible that during short periods of instability a hip strategy may be employed in a single burst of less than half a second to return balance to a region preferred by an ankle strategy. Future research in this area may wish to explore different time windows, or employ other methods with improved time-frequency resolution, such as wavelet analysis.

In summary, the percentage of time spent in different control strategies was determined for perturbed and unperturbed balance in standing and handstand. During both perturbed and unperturbed balance, the prevalent control strategies were an ankle strategy in standing and a wrist strategy in handstand. Findings reveal that the CNS maintains balance during a variety of tasks and postures by employing an individual control strategy. This strategy can be described as a single segment inverted pendulum control model, controlled by torque about the most inferior joint accompanied by synergistic torques about superior joints.

## Supplementary Material

Statistical Results

## Data accessibility

The ethical approval awarded by our institution permits data to be stored on an open repository only if this is explicitly declared on the subject information sheet and consent form. This was not the case for this particular study as elite national and international level gymnasts were recruited and could be identified from the raw data. All data from this study is stored on the private repository of the Biomechanics and Motor Control group at Loughborough University, and access to these data are available through the corresponding author. The electronic supplementary data included with this article contains statistical results for all comparisons between conditions.
