# Supplementary material for: Balance control strategies during perturbed and unperturbed balance in standing and handstand
Source: R Soc Open Sci. 2017 Jul 26;4(7):161018. doi: 10.1098/rsos.161018 (PMC5541526; doi:10.1098/rsos.161018)
Supplement: Statistical Results [file rsos161018supp1.doc]

**Table S1:** The percentage of time spent in each control strategy in standing (ST) and handstand (H) for unperturbed balance with eyes open (EO) and eyes closed (EC).

|  | Primary | Secondary | Tertiary | Mixed | Non-significant |
| --- | --- | --- | --- | --- | --- |
|  | Mean ± SD | Mean ± SD | Mean ± SD | Mean ± SD | Mean ± SD |
| Standing (EO) | 93.5 ± 4.2 | 1.9 ± 1.9 | 0.5 ± 0.6 | 0.1 ± 0.2 | 4.0 ± 2.1 |
| Standing (EC) | 96.9 ± 2.7 | 0.9 ± 1.1 | 0.2 ± 0.3 | 0.0 ± 0.0 | 2.0 ± 1.5 |
| Handstand (EO) | 91.4 ± 11.2 | 2.9 ± 3.6 | 0.3 ± 0.6 | 1.7 ± 3.2 | 3.6 ± 4.7 |
| Handstand (EC) | 85.1 ± 15.1 | 5.4 ± 6.5 | 0.9 ± 1.7 | 2.4 ± 4.3 | 5.9 ± 5.3 |
| Two-way ANOVA: | | | | | |
| Interaction | **25.5****** | **9.7*** | **5.1*** | 1.7 | **22.7****** |
| Vision | 1.5 | 1.2 | 1.1 | 0.9 | 0.1 |
| Posture | 2.9 | 2.8 | 0.8 | 3.4 | 1.4 |
| Multiple t-tests: | | | | | |
| Eyes Open: ST vs. H | 0.5 | -0.7 | 2.1 | -1.7 | 0.2 |
| Eyes Closed: ST vs. H | **2.6*** | **-2.2*** | -1.5 | -1.9 | **-2.6*** |
| Standing: EO vs. EC | **-3.2**** | **2.6*** | **2.8*** | 1.4 | **3.0*** |
| Handstand: EO vs. EC | **3.4**** | -2.2 | -1.9 | -1.1 | **-3.7***** |

Note: Significant t-tests are indicated by * (p < 0.05), ** (p < 0.01), *** (p < 0.005), and **** (p < 0.001); primary strategies are wrist (handstand) and ankle (standing), secondary strategies are elbow (handstand) and hip (standing), tertiary strategies are shoulder (handstand) and knee (standing).

**Table S2:** The percentage of time spent in each control strategy in standing (ST) and handstand (H) for perturbed trials: forwards small (Fs), forwards large (Fl), backwards slow (Bs) , and backwards large (Bl).

|  | Primary | Secondary | Tertiary | Mixed | Non-significant |
| --- | --- | --- | --- | --- | --- |
|  | Mean ± SD | Mean ± SD | Mean ± SD | Mean ± SD | Mean ± SD |
| Standing – Bl | 93.0 ± 6.5 | 1.4 ± 2.7 | 0.1 ± 0.5 | 0.0 ± 0.0 | 5.4 ± 5.9 |
| Standing – Bs | 93.9 ± 8.1 | 2.5 ± 5.7 | 0.2 ± 0.4 | 0.0 ± 0.0 | 3.4 ± 4.2 |
| Standing – Fl | 87.7 ± 10.5 | 2.2 ± 3.1 | 1.4 ± 2.4 | 0.0 ± 0.0 | 8.7 ± 7.2 |
| Standing – Fs | 87.7 ± 13.0 | 2.9 ± 4.8 | 2.0 ± 3.6 | 0.9 ± 2.1 | 6.5 ± 6.7 |
| Handstand – Bl | 90.8 ± 6.4 | 3.7 ± 3.9 | 0.0 ± 0.0 | 0.0 ± 0.0 | 5.0 ± 3.4 |
| Handstand – Bs | 83.1 ± 11.1 | 6.5 ± 7.2 | 0.2 ± 0.3 | 1.3 ± 2.5 | 6.3 ± 4.3 |
| Handstand – Fl | 61.5 ± 16.9 | 17.8 ± 12.9 | 0.8 ± 2.6 | 1.8 ± 5.9 | 17.7 ± 8.9 |
| Handstand – Fs | 67.7 ± 20.0 | 14.9 ± 12.8 | 2.9 ± 7.2 | 2.9 ± 6.1 | 11.2 ± 7.4 |
| Two-way ANOVA: | | | | | |
| Interaction | **4.8**** | **4.4*** | 0.4 | 0.6 | 2.4 |
| Perturbation | **11.3****** | **6.9***** | 2.0 | 1.6 | **9.7****** |
| Posture | **30.1****** | **14.8***** | 0.0 | 2.3 | **7.4*** |
| Multiple t-tests: | | | | | |
| Backward large: ST vs. H | 0.8 | -1.6 | 1.0 | 0.0 | 0.2 |
| Backward small: ST vs. H | **2.9*** | -1.2 | 0.0 | -1.7 | -1.6 |
| Forwards large: ST vs. H | **4.6***** | **-4.0***** | 0.5 | -1.0 | **-2.5*** |
| Forwards small: ST vs. H | **3.1*** | **-2.8*** | -0.6 | -1.2 | -1.8 |
| Standing: Bl vs. Bs | -0.3 | -0.5 | -0.1 | 0.0 | 1.0 |
| Standing: Bl vs. Fl | 1.4 | -0.7 | -1.6 | 0.0 | -1.0 |
| Standing: Bs vs. Fs | 1.6 | -0.2 | -1.6 | -1.4 | -1.5 |
| Standing: Fl vs. Fs | 0.0 | -0.4 | -0.6 | -1.6 | 0.9 |
| Handstand: Bl vs. Bs | 2.1 | -1.5 | -1.5 | -1.7 | -0.7 |
| Handstand: Bl vs. Fl | **5.0****** | **-3.3**** | -1.0 | -1.0 | **-4.6***** |
| Handstand: Bs vs. Fs | **2.9*** | **-2.5*** | -1.3 | -0.8 | **-2.9*** |
| Handstand: Fl vs. Fs | -1.2 | 0.7 | -0.9 | -1.1 | **2.7*** |

Note: Significant t-tests are indicated by * (p < 0.05), ** (p < 0.01), *** (p < 0.005), and **** (p < 0.001); primary strategies are wrist (handstand) and ankle (standing), secondary strategies are elbow (handstand) and hip (standing), tertiary strategies are shoulder (handstand) and knee (standing).
